# A reflection on “Intriguing aspects of lanthanide luminescence”

**DOI:** 10.1039/d5sc90251g

**Published:** 2025-12-04

**Authors:** Svetlana V. Eliseeva

**Affiliations:** a Centre de Biophysique Moléculaire, CNRS UPR 4301, Université d'Orléans Orléans 45071 France svetlana.eliseeva@cnrs-orleans.fr

## Abstract

Lanthanide luminescence surrounds us in everyday life and is essential for cutting-edge technological and healthcare applications. In 2013, we published a minireview in *Chemical Science* (*Chem. Sci.*, 2013, **4**, 1939–1949, https://doi.org/10.1039/C3SC22126A) that was focused on the latest innovations and less-known aspects of lanthanide luminescence in applications such as biological imaging, sensing and therapy, security inks and anti-counterfeiting tags, luminescent probes and sensors, and solar energy conversion. Herein, a discussion of the progress of this field is provided with support of the most recent examples in the areas of circularly polarized luminescence, nanothermometry and multiplexed biological imaging in the extended second near-infrared window (NIR-II, 1000–3000 nm).

## Abstract 2

In celebration of our 15th anniversary and some of our most popular articles, Svetlana V. Eliseeva reflects on the area of lanthanide luminescence, following on from a minireview published in *Chemical Science*: https://doi.org/10.1039/C3SC22126A.

## Graphical abstract

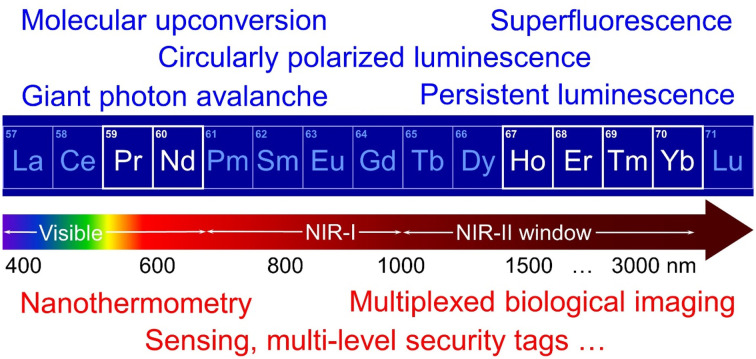

## Scope

The specific [Xe]4f^*n*^ (*n* = 0–14) electronic configuration of trivalent lanthanide ions (Ln^III^), the inner character of the 4f orbitals and the forbidden nature of most f–f transitions are responsible for their unique luminescence properties, namely, fingerprint emission bands in the ultraviolet (UV), visible and near-infrared (NIR) ranges, minimally affected by the experimental conditions, and long luminescence lifetimes. Ln^III^-based materials find widespread practical and now commercialized applications as phosphors for lighting and light-emitting diodes; fibre amplifiers for telecommunications; laser systems that can work in space and on earth for sensing and surgical procedures; scintillators for medical imaging, image-based inspection, well logging, security and radiation safety; anti-counterfeiting and security tags for banknotes, official documents and unambiguous authentication of genuine items; energy convertors for solar cells; and plant growth acceleration films for future green houses.^1^

The main objective of this Commentary is to follow up on the main topics and perspectives discussed in the minireview entitled “Intriguing aspects of lanthanide luminescence”, which was published in 2013 in *Chemical Science* (https://doi.org/10.1039/C3SC22126A).^2^ In the latter, we have highlighted the latest innovations and less-known, at that time, aspects in the field of Ln^III^ luminescence. More specifically, the use of molecular probes and conjugates, and upconverting (UC), downconverting (DC), downshifting (DS) and persistent luminescence (PersL) nanoparticles (NPs) for biological imaging, sensing and therapy, security inks and anti-counterfeiting tags, luminescent probes and sensors, and solar energy conversion has been presented. Since 2013, all of these application fields, supported by tremendous efforts in fundamental research, artificial intelligence (AI) and theoretical methods, have undergone remarkable progress, allowing dreams about qubits and quantum computing,^3^ or self-powered wearable and implantable point-of-care devices for health monitoring and drug delivery^4^ to become a reality. The field of Ln^III^ luminescence remains very attractive, dynamic and regularly reviewed, with >2000 publications and >100 reviews per year on this topic in the last five years according to the Web of Science. The cutting-edge discoveries during the last decade and eye-catching applications of Ln^III^ luminescence in solid-state lighting, photovoltaics, mechanoluminescent probes and the biomedical field have just been highlighted in a freshly released review article.^5^ Therefore, in this Commentary, along with a brief follow up on the progress of UC and PersL NPs, the priority will be given to the most recent examples, touching areas such as circularly polarized luminescence, nanothermometry and biological imaging in the extended second NIR window (NIR-II, 1000–3000 nm), including the multiplex approach.

## Discussion

The advantages of Ln^III^ luminescence for biological imaging and sensing have been widely recognized since the commercialization of DELFIA (dissociation enhanced lanthanide fluorescence immunoassay) in 1980s. Subsequently, many Ln^III^ complexes with derivatives of polyaminocarboxylates, dipicolinates, β-diketonates, podate-based and macrocyclic ligands, as well as polymetallic structures including dendrimers, coordination polymers and metal–organic frameworks (MOFs), inorganic, silica and polymer NPs have been designed and tested for this purpose.^6^ The main objectives have been the improvement of sensitivity and selectivity of detection, and the design of responsive imaging agents able to recognize several analytes at the same time and to perform dynamic monitoring of biological processes *in vitro* and *in vivo*. In the last decade, the development of microfluidic technologies allowed significant reduction of the time of analysis and the amount of the reagents used while providing very sensitive and real-time read-outs in wearable or implantable point-of-care devices.^5,7^ Additionally, commercialization and miniaturization of detectors operating in the NIR-II range have generated an urgent quest for Ln^III^-based compounds exhibiting emission in this imaging window due to the significant advantages that it provides (see below).

**Fig. 1:**
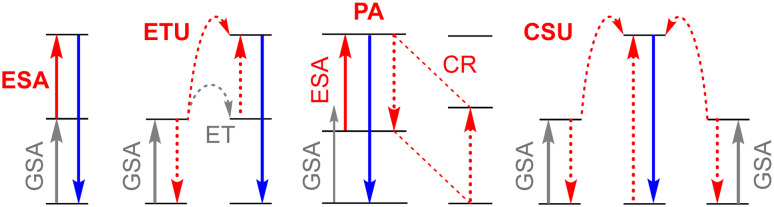
Principle upconversion mechanisms in Ln^III^-based compounds. From left to right: excited-state absorption (ESA), energy transfer upconversion (ETU), photon avalanche (PA), cooperative sensitization upconversion (CSU); GSA stands for ground-state absorption, CR for the cross-relaxation and ET for energy transfer.

Upconversion is a nonlinear anti-Stokes phenomenon that allows conversion of two or more low-energy photons into higher-energy ones through different types of mechanisms (Fig. 1).

During the last decade the field of Ln^III^-based UC materials has expanded enormously.^8^ Fundamental research has provided significant insights into the UC mechanisms, allowing establishment of the role of the size, crystal phase, core–shell structure, defects and size of Ln^III^, and making the tailored design of UC materials possible. To minimize surface effects, boost the brightness of UCNPs and overcome the low absorption of f–f transitions, different strategies of surface functionalization with passivating agents/layers, organic molecules or transition metal complexes have been developed. The use of surface plasmon resonance to enhance UC has also been explored. A recently described alternative approach to enhance the emission efficiency of UCNPs is to use dual-wavelength co-excitation.^9^ An enhancement of up to 800% has been demonstrated for NaYF_4_:Yb^III^,Tm^III^ sensitized using a combination of 975 nm and 1732 nm lasers. Synthetic strategies have also been improved, and uniform NPs with controlled size and desired multi-layer structure can now be reproducibly obtained. Initial studies in the field of Ln^III^-based UCNPs have mainly been devoted to Yb^III^/Er^III^ systems in which Yb^III^ acts as an activator and its excitation at ∼980 nm leads to the sensitization of Er^III^ (acceptor) emission in the visible range. Today, dictated by society’s need for innovative security solutions, detection and therapeutic approaches, and energy-saving technologies to make our world more sustainable, the range of Ln^III^ ions and combinations thereof used to create UC materials has been extended to the whole palette enabling emissions ranging from UV through visible and NIR. The generation of UV light upon NIR excitation in UCNPs allows the release of drugs or generation of reactive oxygen species *in vivo* for enhanced phototherapies.^10^ Full control over the UC emissions of Er^III^ and Tm^III^ in specifically designed multilayer core–shell NPs, NaYF_4_:Yb^III^/Tm^III^@NaYF_4_@NaYbF_4_:Er^III^/Tm^III^@NaYF_4_, has been achieved by simply altering the excitation modes of Yb^III^ at 980 nm (continuous-wave, short-pulse or time-gating). Colour tuning in the entire visible range has been demonstrated, opening perspectives for emerging photonic applications including volumetric displays.^11^

**Fig. 2:**
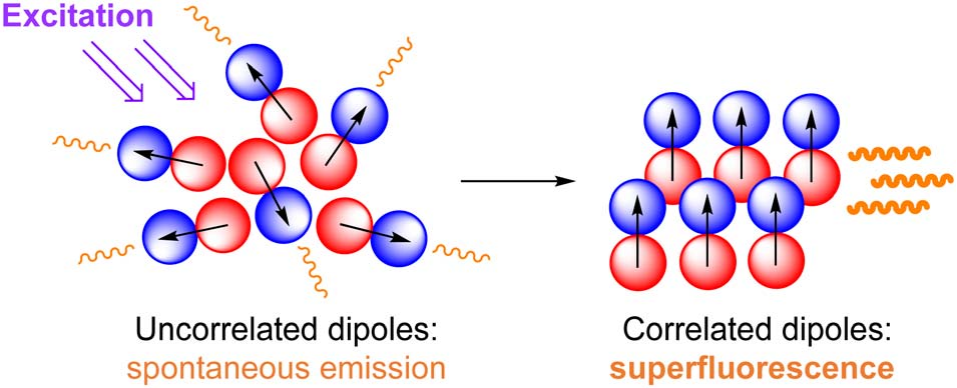
Main principle of superfluorescence.

A superfluorescence (SF, Fig. 2) phenomenon in UCNPs was documented in 2022.^12^ This quantum phenomenon is induced by an ultra-short high-power laser excitation pulse and leads to a coherent coupling of several Ln^III^ ions within a single UCNP. In turn, a huge shortening of the Ln^III^ emission lifetime is observed, allowing the achievement of superior upconversion efficiency. SF UCNPs are considered as a second-generation quantum technology and are expected to be used in advanced imaging and sensing applications.

The discovery of photon avalanche (Fig. 1), a highly nonlinear phenomenon, in UCNPs has opened new avenues for applications in super-resolution microscopy (nanoscopy), as well as in ultra-sensitive sensing, quantum optics and optical switching. An exceptional optical nonlinearity of a factor of ∼150 in 27 nm NaLuF_4_:15% Tm^III^ NPs has been induced by sublattice reconstitution, while further expansion of a photon-avalanche network in 176 nm nanodiscs has led to an extreme nonlinearity factor of ∼500.^13^ As a result, the use of NaLuF_4_:15% Tm^III^ nanocrystals allowed lateral and axial resolutions of ∼33 nm and ∼80 nm, respectively, to be obtained in 3D imaging experiments.

Despite associated challenges, in the last decade, since the demonstration of the sensitization of Er^III^ green emission at 540 nm upon excitation of Cr^III^ at 750 nm in a hetero-trinuclear triple-stranded helicate complex in 2011,^14^ there has been growing interest in Ln^III^ molecular systems that exhibit UC luminescence.^15^ Tailored design of Ln^III^ complexes with organic ligands, supramolecular assemblies, Ln^III^ molecular clusters and metal–organic cages allowed the observation of UC luminescence of not only Er^III^, but also Ho^III^, Tb^III^ and Eu^III^, using Cr^III^ or Yb^III^ as activators. Ln^III^ UC can also be used to sensitize the emission of organic dyes or d-transition metals. Despite these promising results, the efficiency of UC luminescence in Ln^III^ molecular compounds remains low, hindering their practical applications. Nevertheless, recently, UC emission of Tb^III^ could be sensitized in an aqueous solution of the Yb^III^/Tb^III^ heteropolynuclear complex, with a quantum yield comparable to the values reported for Er^III^-based molecular systems in organic solvents.^16^ Additionally, for the first time, green UC emission of Tb^III^ in a Yb^III^/Tb^III^ complex incorporated into polymeric nanoparticles has been used for living-cell imaging upon sensitization of Yb^III^ at 980 nm.^17^

**Fig. 3:**
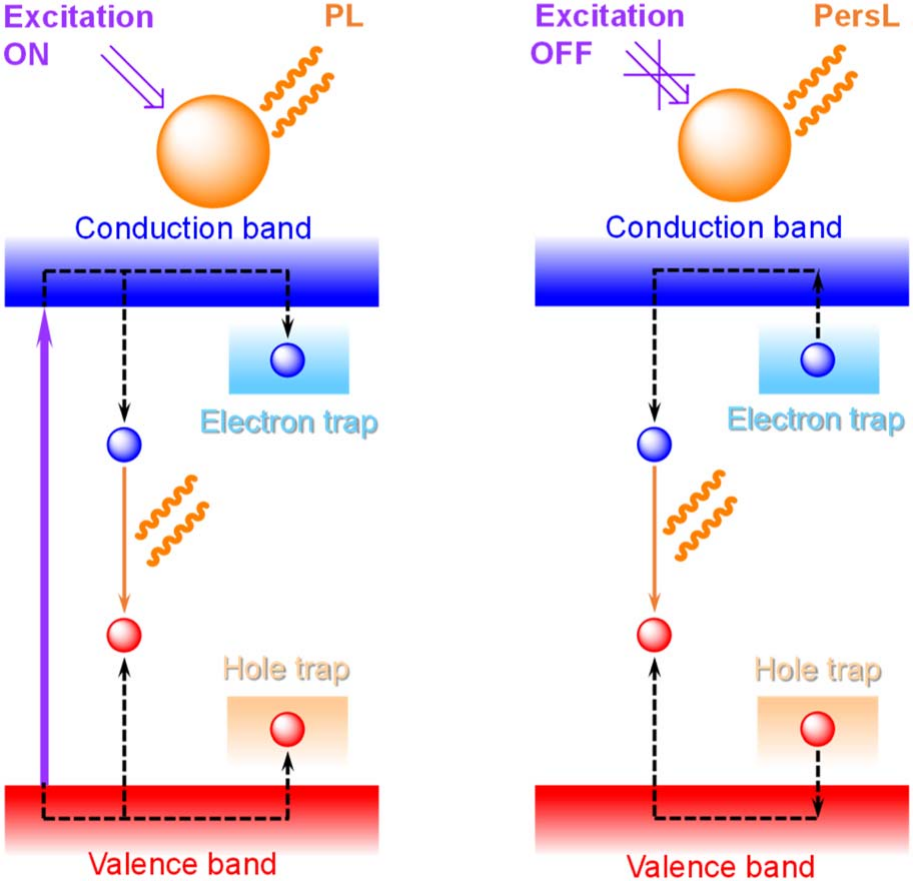
Simplified mechanism of persistent luminescence (PersL) in inorganic materials upon photoexcitation; PL stands for photoluminescence.

Persistent luminescence (PersL, Fig. 3) is a property of materials to store light energy and retain emission for minutes or hours after the switch off of excitation.^18^ It is most often activated by UV light, but NIR or X-rays^19^ can also be used. PersL holds great promise for bioimaging and sensing applications, particularly in the NIR-II range (see below), since it is not affected by the background autofluorescence, allowing images to be obtained with significantly higher contrast and resolution. During the last decade, increasing attention has been given to the development of Ln^III^-based NPs, including UCNPs, that exhibit PersL. Synthetic approaches that do not require post-annealing, while enabling isolation of uniform NPs with active surface for further functionalization, *e.g.*, sol–gel processing, the template method, solvo- or hydrothermal treatment and pulsed laser ablation, have been developed. To enhance Ln^III^ PersL in NPs surface passivation, dye sensitization and surface plasmon resonance have been explored. As a result, monodisperse PersLNPs with controlled size that exhibit tuneable emission colours can now be obtained and used for multi-level anti-counterfeiting, optical data storage and encoding, biosensing and imaging.^20^

**Fig. 4:**
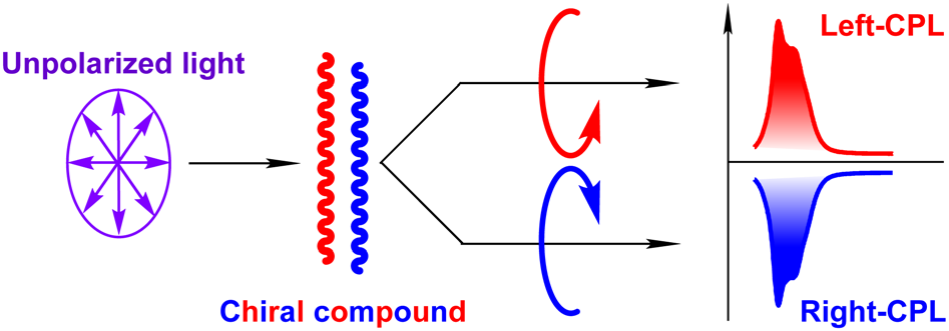
Schematic presentation of circularly polarized luminescence (CPL).

In the last decade, there has been increasing interest in designing and studying Ln^III^-based compounds that exhibit circularly polarized luminescence (CPL, Fig. 4).^21^ CPL can provide additional advantages for a variety of emerging applications in modern technologies including 3D displays, advanced information storage and encryption, quantum communication, anti-counterfeiting tags with an extra level of security, molecular fingerprinting and enantioselective imaging. The latter has become possible due to the development of the corresponding instrumentation and methods, including CPL laser scanning confocal microscopy.^22^ CPL is characterized by the differential emission of left- and right-handed circularly polarized light from chiral systems and is quantitatively characterized by a dissymmetry factor (*g*_lum_). Several strategies to design Ln^III^-based chiral compounds with improved values of *g*_lum_ have been suggested. Ln^III^ complexes with chiral ligands possessing improved rigidity, exhibiting aggregation-induced emission or electrostatic interactions with DNA, have been studied. Chirality can also be induced by the incorporation of achiral systems into chiral environments like liquid crystals, carbon nanotubes or MOFs. Initial studies have mainly been devoted to Eu^III^ and Tb^III^ chiral systems, while now, the whole range of Ln^III^ emissions can be obtained, including the ones exhibiting CPL in the NIR range, *e.g.*, Yb^III^, Nd^III^ and Er^III^. CPL in most of the reported examples of Ln^III^-based compounds can be sensitized upon excitation in the UV range, which can be detrimental for biological systems. To shift the excitation to the NIR range, two-photon excited CPL has been successfully demonstrated in tailored Eu^III^ complexes with ligands comprising a pyridine bis-oxazoline (PyBox) core conjugated through the pyridine 4-position with a phenylacetylene unit. Alternatively, upconversion has been coupled with CPL by incorporating UCNPs into chiral hosts (helical nanotubes, cellulose nanocrystals or liquid crystals) or by designing supramolecular tetrahedral complexes.^5^

The area of Ln^III^-based luminescent nanothermometers (LNThs) has attracted exponentially increasing attention during the last decade.^23^ Nanothermometry allows a non-invasive assessment of the temperature of submicron systems *via* spectral or temporal analysis of the emitted light. Precise evaluations of temperature variations in biological systems can serve as diagnostic indicators of different physiological processes as well as pathologies including neurologic and cardiovascular diseases or injuries, cancer, and inflammations. Nanothermometers that can allow monitoring of temperature with high spatial resolution and operate at cryogenic temperatures are essential for advancing quantum technologies and superconducting systems. Ln^III^-based NPs and MOFs are the most commonly used compounds to design LNThs, although they can also be created by taking advantage of molecular systems, like metallacrowns.^24^ As a recent example, core–shell NaYF_4_:Yb^III^/Ho^III^@NaYF_4_:Yb^III^/Pr^III^ nanocrystals with an optimized concentration ratio and spatial distribution of Ho^III^ and Pr^III^ have enabled ultrasensitive temperature sensing in the NIR-II range with a maximum relative sensitivity (*S*_r_) of 2.03% K^−1^.^25^ In addition to DS luminescence, these NPs also exhibit UC emission, which makes them attractive for multimodal anti-counterfeiting applications.

**Fig. 5:**
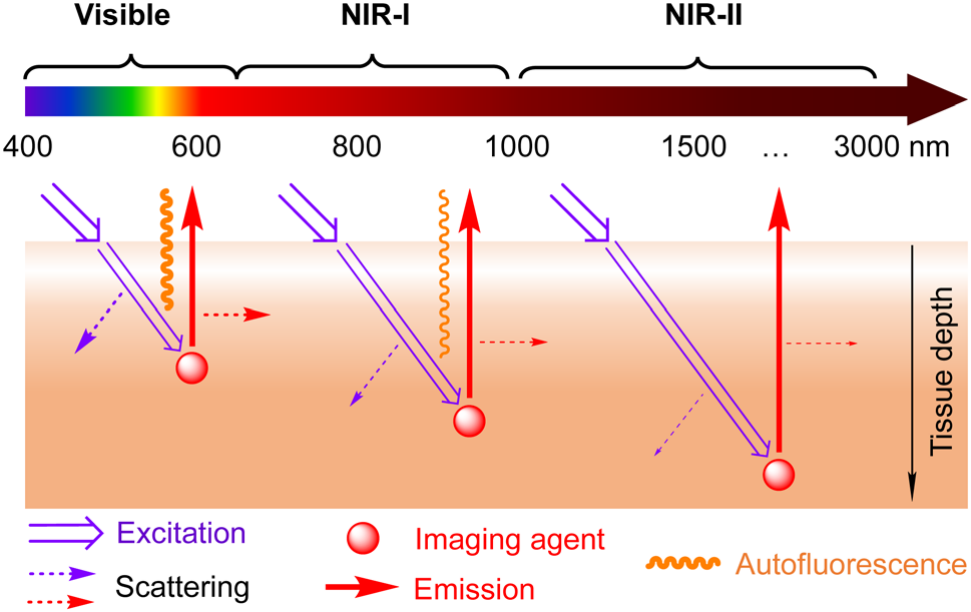
Schematic presentation of advantages provided by optical imaging in the NIR-II window: deeper penetration depth, lower scattering and minimized autofluorescence.

Optical imaging in the NIR-II window (Fig. 5) has emerged as a powerful tool to monitor biological processes *in vivo* by providing high resolution and contrast from deep tissues due to reduced photon scattering and minimal autofluorescence. During the last 15 years, this approach has revolutionized the field of biomedical imaging, allowing *in vivo* molecular imaging and image-guided surgery of tumours, and dynamic monitoring of vascular structures, lymph nodes, and skeletal, gastrointestinal and cardiovascular disorders.^26^

**Fig. 6:**
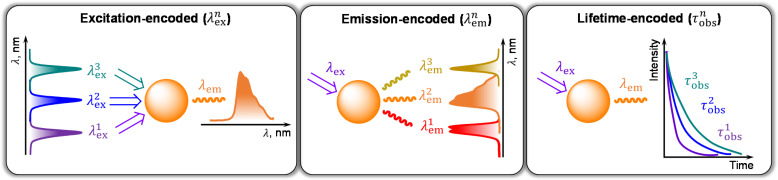
Main strategies of multiplexed optical imaging.

Additionally, multiplexed NIR-II imaging (Fig. 6) enables the simultaneous detection of several analytes and real-time observation of different physiological or pathological processes, which is essential to understand and effectively access complex biological mechanisms *in vivo* without ambiguity in interpretation.^27,28^ Ln^III^ emissions in the NIR-II range from UCNPs doped with Tm^III^, Er^III^ or Ho^III^ as activators can be generated upon laser excitations at 1870/1710 nm, 1532 nm or 1208 nm.^29^ Of particular promise is the use of Tm^III^-doped UCNPs since in this case the excitation wavelength does not overlap with strong absorption bands of water located at 1200 nm, 1445 nm or 1920 nm, avoiding limitations associated with lower excitation efficiency and penetration depths.

Core–shell NaYF_4_:Yb^III^/Ln^III^@NaYF_4_ DSNPs were first used for multispectral NIR-II imaging *in vivo* in 2013.^30^ Depending on the nature of the co-doped Ln^III^, emissions at 1185 nm (Ho^III^), 1310 (Pr^III^), 1475 (Tm^III^) and 1525 (Er^III^) nm have been monitored in mice upon stimulation of Yb^III^ at 980 nm. In 2023, in order to expand the number of emission channels available for NIR-II imaging, the possibility of generating emissions of Tm^III^ at 1852 nm and 2324 nm as well as Ho^III^ at 2030 nm and 2842 nm, upon excitation at either 980 nm or 808 nm in NaYbF_4_:Ln^III^@NaYbF_4_@NaYF_4_ NPs with optimized structure, was demonstrated.^31^ Such NPs have been successfully used for dynamic multiplexed imaging of vascular and lymphatic systems in intestines and tumours.

A recent breakthrough discovery is the demonstration of six-channel NIR-II *in vivo* imaging using Ho^III^-doped NPs that can be excited at 1143 nm and exhibit tuneable emissions in the range from 1000 nm to 2200 nm, controlled by the nature of the co-doped Ln^III^.^32^

Nanothermometry in the NIR-II range is also important for the non-invasive monitoring of temperature from deep tissues. Thus, ratiometric LNThs that combine PbS quantum dots with NaYbF_4_:2% Er^III^,2% Ce^III^@NaYF_4_:10% Yb^III^@NaYF_4_:40% Nd^III^ DSNPs, and exhibit a thermally sensitive emission at 1280 nm along with an Er^III^ signal at 1525 nm under 808 nm excitation, have been used for the contactless monitoring of cerebral lesion temperatures in mice with ischemic stroke.^33^

X-ray activated NaY(Gd)F_4_ PersLNPs with a core–shell structure doped with Nd^III^, Ho^III^, Tm^III^ or Er^III^ exhibit narrow emission bands in the NIR-II range at 1064 nm, 1180 nm, 1475 nm or 1525 nm, respectively, and through different injection pathways have been successfully used for multiplex NIR-II imaging of blood vessels, tumours and visceral organs (lung, liver and spleen) in living mice.^19^ Furthermore, to enhance PersL and overcome the challenge of its fading with time, optothermal stimulated persistent luminescence and therapy (OSPLIT) NPs have been designed.^34^ They consist of a luminescent core (NaYF_4_:3% Er^III^@NaGdF_4_) and a photothermal shell (NaNdF_4_@Prussian blue). Er^III^ emission at 1525 nm in OSPLIT NPs is initiated by X-rays, while further laser irradiation at 808 nm converts the excitation energy into heat and allows NIR-II PersL to be boosted through the release of charge carriers in the core. The OSPLIT approach enables not only high contrast NIR-II imaging *in vivo* but also the thermal ablation of lymph node metastases, providing a therapeutic effect.

The difference in Ln^III^ luminescence lifetimes can also be used for multiplexing in the NIR-II range (Fig. 6, right).^27^ For example, by varying the thickness of an Yb^III^-doped energy-relaying layer (NaYF_4_:Yb^III^) between the outer NaNdF_4_:Yb^III^ light-absorbing shell (808 nm) and the Yb^III^/Ln^III^ (Ln^III^ = Nd^III^, Ho^III^, Pr^III^, Tm^III^, Er^III^) co-doped activation layer (NaGdF_4_:Yb^III^,Ln^III^) it is possible to modulate NIR-II luminescence lifetimes within up to 3-orders of magnitude in the case of Ln^III^ = Er^III^.^35^ To demonstrate the validity of the lifetime-encoded multiplexed approach, synthesized NPs have been conjugated with three primary antibodies against breast cancer biomarkers and used for non-invasive diagnostic *in vivo*.

For NIR-II imaging agents based on Ln^III^ molecular compounds, in addition to the luminescence quenching in aqueous environments induced by the overtones of O–H vibrations, it is necessary to minimize the effect of C–H bonds largely present in organic ligands. In 2013, we mentioned a novel class of luminescent Ln^III^ complexes, Ln^III^/Zn^II^ metallacrowns (MCs) with an “encapsulated sandwich” structure, as efficient scaffolds able to protect and efficiently sensitize NIR-emitting Ln^III^, boosting their quantum yields.^36^ Since that time, rationalization approaches have been developed to tune the excitation wavelengths, quantitative photophysical parameters and water solubility of such systems.^37^ Moreover, a second generation of luminescent Ln^III^/M^III^ (M^III^ = Ga^III^, Al^III^, Cd^III^) MCs with a variety of crystal structures has emerged.^38^ The functionalization of Ln^III^/Ga^III^ MCs with organic chromophores or d-transition metal complexes demonstrated their applicability for NIR-II living cell imaging^39^ and high potential for multiplexed detection in the NIR-II window.^40^

Another class of Ln^III^ molecular compounds that possesses superior photophysical characteristics in the NIR-II region is sandwich-type Ln^III^-tetrapyrrole complexes with Kläui's tripodal ligands.^41^ In a very recent example, a series of Er^III^ phthalocyanine complexes, termed “Lanbow”, has been created.^42^ Tailored ligand design in Lanbow has allowed the achievement of tuneable (in the range of 670–790 nm) absorption bands able to sensitize NIR-II Er^III^ emission centred at 1530 nm. These complexes have been formulated into micelles using Pluronic F127 poloxamer and used in excitation-encoded (Fig. 6, left) real-time *in vivo* imaging in live mammals. Furthermore, the application of deep-learning protocols allowed automated analysis of images, enabling multiparametric fluorescence-guided surgery.

## Conclusions and perspectives

Definitively, the bright future promised in 2013 for Ln^III^-based compounds became a reality. Tailored design combined with theoretical approaches and innovative synthetic methodologies allowed significant improvement of functional properties of Ln^III^-based nanomaterials and molecular compounds and the successful use of them for anti-counterfeiting, NIR-II multiplexed imaging and therapy, fluorescence-guided surgery and nanothermometry. Discovery of a superfluorescence phenomenon and an extreme photon avalanche for UCNPs opens ways to second-generation quantum technology, ultra-sensitive and super-resolution approaches.

However, the commercialization of new technologies is often very limited by financial aspects of practical implementations that have to balance inputs and potential benefits. Nevertheless, several companies (*e.g.*, Honeywell Lumilux® portfolio, Olnica) are now using Ln^III^-based compounds as safety and security tags.

On the other hand, despite significant progress, the use of Ln^III^-based imaging agents for *in vivo* applications and their translation to pre-clinical and clinical studies is still limited by several challenges. Most of the nanomaterials are still >10 nm, causing possible toxicity concerns.^43^ To remove the fading-time limitation for *in vivo* imaging, PersLNPs that allow recharging over multiple cycles using NIR light or low-dose X-rays should be developed. Molecular upconverting and NIR-II Ln^III^-based imaging agents still have limited efficiency and need to be encapsulated into nanoparticles or formulated into micelles. LNThs are affected by the presence of bias (additional environmental conditions, *e.g.*, pH, viscosity, ionic strength, biomolecules) that reduces the reliability of the thermal readout.^44^

There are several other points that should be considered to continue the advancement of the field of Ln^III^ luminescence and to support the initiative to make our world more sustainable. To accelerate rationalization procedures aimed at tailored designs of Ln^III^-based compounds or at predictions of toxicity by taking advantage of the rapidly developing field of AI and deep learning protocols,^45^ we have to establish standardized procedures for the characterization of functional properties and, in particular, for the determination of photophysical parameters and toxicity evaluation of UCNPs, PersLNPs and LNThs. This would, for example, enable performing cross-comparison of their performance between different publications.

The development of sustainable green chemistry methods for the synthesis of Ln^III^-based compounds would minimize the impact on the environment. Additionally, innovative synthetic procedures allowing precise control over the formation of nanosized metal–organic complexes in confined reactors, like liposomes, should be considered.^46^ New classes and design strategies of nanosized and molecular Ln^III^ luminescent compounds are also very welcome.

Further advancements in the field of Ln^III^-based compounds that exhibit CPL in the NIR-II range using biologically-friendly excitation wavelengths will open new perspectives for the selective recognition and tracking of chiral molecules *in vivo*. Attention should be given to studies of compounds that display CPL induced by a magnetic field. Such systems do not require the use of chiral ligands/hosts and intricate designs, and have already shown great potential in the field of sensitive CPL thermometers, for example.^47^

The design of NIR-II imaging agents that would be selectively delivered to the target of interest or activated by the microenvironment (pH, hypoxia, enzymes) would minimize side effects and allow the dynamic monitoring of physiological processes, progression of diseases and effects of drugs, enabling better understanding of the mechanisms *in vivo*, and the development of new therapeutic approaches. Multifunctional assemblies that will respond to different stimuli (light, force, sound, temperature), CPL photoswitches, or NIR-II imaging agents that can be used in other imaging modalities (photoacoustic, magnetic resonance imaging, tomography) or exhibit therapeutic effects, will open new perspectives for cutting-edge applications.

Altogether, to further advance the field of Ln^III^ luminescence and its impact on high-technological applications and healthcare solutions, mutual contributions from chemists, physicists, biologists, medical doctors and engineers are required. Such interdisciplinary efforts should accelerate translation of existing innovative approaches to real practical applications and facilitate new discoveries of unlimited possibilities provided by Ln^III^ luminescence.

## Author contributions

S. V. E. wrote the article.

## Conflicts of interest

There are no conflicts to declare.

## Data Availability

No primary research results, software or code have been used in this Commentary.
